# Validity and reliability of Turkish version of the Adult Eating Behaviour Questionnaire for adult participants

**DOI:** 10.1017/S1368980023001751

**Published:** 2023-11

**Authors:** Hülya Yardımcı, Nursena Ersoy, Nazlı Nur Aslan Çin

**Affiliations:** 1 Department of Nutrition and Dietetics, Ankara University, Ankara, Turkey; 2 Department of Nutrition and Dietetics, Karadeniz Teknik University, Trabzon, Turkey

**Keywords:** Eating behaviour, BMI, Appetitive traits, Obesity

## Abstract

**Objective:**

The aim of this study is to assess the validity and reliability of the Adult Eating Behaviour Questionnaire (AEBQ-TR) for adults.

**Design:**

Hunot et al. (2016) developed the original questionnaire, which was modified and translated into Turkish. On data collected from adults, construct validity was assessed using exploratory factor analysis and confirmatory factor analysis. Pearson’s and Cronbach’s correlation coefficients were used to evaluate reliability and validity (*P* < 0·05).

**Setting:**

This research was carried out in Ankara, Turkey.

**Participants:**

A total of 311 adults from Ankara (148 men and 163 women) took part in the study. Seventy-two of these adults take the retest.

**Results:**

In the present study, 311 adults with a mean age of 29·3 ± 11·3 years participated. Factor loadings ranged from 0·404 to 0·907. In general, food approach and food avoidance scales showed a positive correlation within themselves. According to the results of confirmatory factor analysis and goodness-of-fit indicators, the seven-factor model showed a better model fit in the Turkish data (chi-square/degrees of freedom = 2·137, root mean error of approximation: 0·061, comparative fit index: 0·884, and normed fit index: 0·850). Higher BMI was associated with higher Emotional Over-eating, higher Enjoyment of the Food, lower Food Satiety and lower Emotional Under-eating.

**Conclusions:**

The Turkish AEBQ is a valid and reliable tool for 20- to 65-year adults to determine appetitive properties related to the aetiology of weight change and especially obesity risk. Besides, AEBQ testing is required for validation in early and late adulthood.

Obesity, characterised by excessive adiposity, is a considerable public health problem whose effects on morbidity and mortality are well known^(1)^. According to the current obesity report of the WHO, 60·0 % of individuals in the European region are overweight or obese^(2)^. According to Eurostat data, the prevalence of obesity is 16·5 % in Europe and 22·3 % in Turkey^(3)^. Both genetic and environmental factors play a considerable role in body weight control^(4)^. In particular, the developments in the food sector have caused delicious and energy-dense foods to become more accessible and cheaper. These changes in the food environment and decreased physical activity in modern life lead to positive energy imbalance and cause body weight gain^(5)^.

The rapid increase in the prevalence of obesity worldwide is generally due to the obesogenic environment^(6)^. Individuals with various genes more responsive to extrinsic food cues or lower sensitivity to satiety have a higher risk of over-eating and obesity in response to the ‘obesogenic’ food environment^(4)^. Changes in eating behaviour as a result of differences in appetite are responsible for the impact of environmental and genetic risks on body weight^(7)^. Regulation of eating behaviour is considered the primary goal for the development of interventions to prevent and treat obesity. Therefore, a better understanding of their aetiology and evolution throughout the life cycle is necessary to establish consistent measures of eating behaviours from childhood to adulthood^(8)^.

In studies examining the relationship between appetite, eating behaviour and body weight in general, valid and reliable questionnaires such as Three-Factor Eating Questionnaire, Dutch Eating Behaviour Questionnaire and Adult Eating Behaviour (AEBQ) were used^(9–11)^. The Three-Factor Eating Questionnaire consists of three sub-dimensions: Cognitive Restraint, Disinhibition and Hunger; Dutch Eating Behaviour Questionnaire consists of Emotional Eating, Restraint and External Eating sub-dimensions^(12,13)^. Unlike these scales, AEBQ examines appetitive features in more detail. The AEBQ, which was adapted from the Child Eating Behavior Questionnaire, includes four ‘food approaches’ (Hunger (H), Food Responsiveness (FR), Emotional Over-eating (EOE), and Enjoyment of Food (EF)) and four ‘food avoidance’ (Satiety Responsiveness (SR), Emotionally Under-eating (EUE), Food Fussiness (FF), and Slowness in Eating (SE)). Unlike AEBQ, the Three-Factor Eating Questionnaire measures emotional eating in the disinhibition sub-dimension. Although Dutch Eating Behaviour Questionnaire measures emotional eating, unlike AEBQ, it examines the effect of emotional state on eating^(14)^.

Currently, the AEBQ is valid in adults and adolescents in the UK, Australia, China, and Mexico in adults, obese adolescents and bariatric surgery patients in the USA and adolescents in Poland^(14–21)^. However, there is no validity and reliability in the Turkish version of this questionnaire in a Turkish-speaking population. Therefore, it is a clear requirement the AEBQ tool is adapted to Turkish society, taking into account cultural and linguistic differences. Therefore, the purposes of this study are (1) to confirm the factor structure of AEBQ-TR, the Turkish version of AEBQ, (2) to determine AEBQ-TR’s both internal and test–retest reliability and (3) to determine whether there is a relationship between AEBQ-TR and appetitive characteristics measured using BMI in the Turkish adult population.

## Material and methods

Data were collected through face-to-face interviews with 311 participants living in Ankara between March 2021 and July 2022. Participants between the ages of 20–65, who can communicate in Turkish, who are not diagnosed with eating disorders, who are not pregnant/lactating, and who do not have chronic diseases, were included in the study. The Ankara University Research Ethics Committee approved the study protocol (protocol number: 56786525–050·04·04/47090), and the Helsinki Declaration principles were applied in the research. Prior to the survey, each participant was informed about the study’s contents and signed an informed consent form, indicating their voluntary participation in the research.

### Turkish adaptation protocol

Andrea Smith’s e-mail communication provided permission to translate the AEBQ. The English version of the AEBQ has been translated into Turkish. The back-and-forth translation method was used to complete the translation. Two translators fluent in English who were unaware of each other translated the questionnaire into Turkish using advanced translation. The two versions were checked, and any inconsistencies were resolved collaboratively by the research team. Another bilingual speaker who did not know the English version translated it back into English. AEBQ content validity was tested by a group of seven nutritionists, two psychologists and a nurse. Experts were asked to rate each question on its simplicity, clarity, relevance and necessity. The research team evaluated the scale with ten people with no major changes, and the questionnaire was finalised after necessary corrections were made.

### Assessment of construct validity

Factor analysis was used to assess the scale’s construct validity. The sample size was determined by multiplying the number of scale items by at least 5–10 times^(22)^. Therefore, at least 175 participants were required to obtain an adequate sample size. Collected data included age, gender, educational status, marital status and BMI. Participants were asked to complete the translated version of the AEBQ. The original questionnaire includes thirty-five questions with a five-point Likert scale (1 = strongly disagree, 5 = strongly agree).

Items in the AEBQ are classified on eight scales, four of which are food approach and four that are food avoidance scales. Hunger, Food Responsiveness, Emotional Over-eating, Enjoyment of the Food scales food approach; Satiety Responsiveness, Emotional Under-eating, Food Fussiness, Slowness in Eating are food avoidance scales. Each scale consists of 3–5 items/questions. In previous validation studies, a better model fit was achieved by removing the eight-factor structure of AEBQ with the Hunger scale or combining it with the Food Responsiveness scale^(14,15,21)^. Subscale scores were calculated using the averages of the items for each scale. Explanatory factor analysis followed the methods used in the original validation study, and varimax rotation principal component analysis was used to test the factor structures of thirty-five questions. The Kaiser–Meyer–Olkin and Bartlett’s sphericity tests were used to determine sample adequacy. Items with factor loadings less than 0·30 or that overlapped were removed from the scale. Internal consistency was assessed using item analysis and the reliability coefficient (Cronbach’s).

### Data analysis

AMOS version 21 was used for confirmatory factor analysis. All statistical analyses were performed using SPSS software version 25.0. Chi-square, root mean error of approximation, comparative fit index and normed fit index were used to assess model fit. Chi-square *P* values greater than 0·05, root mean error of approximation less than 0·08, normed fit index and comparative fit index greater than 0·9 are all acceptable. Explanatory factor analysis investigated the AEBQ’s factorial structure. For the scale’s internal consistency, the standardised parameter Cronbach’s was used. The scale’s test–retest reliability was re-evaluated two weeks later. Besides, simple associations between appetitive trait means and BMI were examined using Pearson’s correlation coefficients. Multivariable linear regression analyses were used to test for associations between BMI and each appetitive trait, adjusting for sex, age, sex, education and marital status as statistical confounders. The statistical significance level was set at *P* < 0·05.

## Results

The sociodemographic features of the individuals participating in the study are shown in Table 1. The mean age of the individuals was 29·3 ± 11·3 years. 69·5 % of individuals are between the ages of 18–29, 52·4 % are women, and 57·9 % have normal BMI. 62·7 % of the participants are high school graduates, and 70·1 % are single. AEBQ-TR was completed a second time by a total of seventy-two participants (36 males; 36 females) aged 31·5 ± 10·7 years.

**Table 1:** Sociodemographic features of participants

	Total (*n* 311)		Test–retest (*n* 72)	
Variable	*n*	%	*n*	%
Age (years)				
Mean	29·3		31·5	
sd	11·3		10·7	
18–29	216	69·5	36	50·0
≥ 30	95	30·5	36	50·0
Sex				
Female	163	52·4	35	48·6
Male	148	47·6	37	51·4
BMI				
Mean	23·9		23·9	
sd	4·4		4·3	
Underweight	26	8·4	3	4·2
Normal	180	57·9	41	56·9
Overweight	73	23·5	21	29·2
Obese	32	10·3	7	9·7
Education				
Primary/Secondary	35	11·3	6	8·4
High school	195	62·7	34	47·2
University/College	81	26·0	32	44·4
Marital status				
Single	218	70·1	41	56·9
Married	93	29·9	31	43·1

### Exploratory factor analysis

Table 2 shows the factor analysis results. Principal component extraction with varimax rotation was used for factor analysis. The established indicators of a high degree of interrelationship between the variables confirmed the relevance of the analysis: Bartlett’s test of sphericity 2 = 5601; *P* = 0·00 and KMO index was 0·83. In this study, thirty-five items in the AEBQ-TR version produced seven factors similar to the original questionnaire. The factor load showing the relationship of each item with the total score was over 0·30, and seven factors explained 62·2 % of the variance. The excluded factors were retained as described in the original article: Three ‘food approach’ and four ‘food avoidance’ scales were among the seven components. Hunger and Food Responsiveness (loaded on a single component), Emotional Over-eating, and Food Enjoyment were the ‘food approach’ scales. Satiety Responsiveness, Emotional Under-eating, Food Fussiness and Slowness in Eating were the four ‘food avoidance’ scales.

**Table 2:** Results of explanatory factor analysis of the AEBQ-TR

Items	Eigenvalue	(% variance explained	Factor loadings*						
			1	2	3	4	5	6	7
			H + FR	SR	EUE	FF	EOE	EF	SE
Q28 = I often feel so hungry that I have to eat something right away	4·22	12·0 %	0·753						
Q32 = I often feel hungry			0·747						
Q6 = I often notice my stomach rumbling			0·619						
Q34 = If my meals are delayed I get light-headed			0·594						
Q22 = I am always thinking about food			0·591						
Q9 = If I miss a meal I get irritable			0·567						
Q13 = I often feel hungry when I am with someone who is eating			0·570						
Q17 = Given the choice, I would eat most of the time			0·537						
Q33 = When I see or smell food that I like, it makes me want to eat			0·452						
Q23 = I often get full before my meal is finished				0·724					
Q31 = I get full up easily	1·34			0·716					
Q11 = I often leave food on my plate at the end of a meal		3·8 %		0·606					
Q30 = I cannot eat a meal if I have had a snack just before				0·461					
Q20 = I eat less when I’m upset					0·859				
Q27 = I eat less when I’m annoyed	3·47	9·9 %			0·837				
Q35 = I eat less when I’m anxious					0·834				
Q18 = I eat less when I’m angry					0·800				
Q15 = I eat less when I’m worried					0·795				
Q19 = I am interested in tasting food I haven’t tasted before	2·64	7·5 %				0·771			
Q7 = I refuse new foods at first						0·722			
Q12 = I enjoy tasting new foods						0·717			
Q2 = I often decide that I don’t like a food, before tasting it						0·666			
Q24 = I enjoy a wide variety of foods						0·622			
Q8 = I eat more when I’m worried	6·96	19·9 %					0·878		
Q10 = I eat more when I’m upset							0·851		
Q5 = I eat more when I’m annoyed							0·825		
Q16 = I eat more when I’m anxious							0·819		
Q21 = I eat more when I’m angry							0·738		
Q1 = I love food	1·49	4·3 %						0·894	
Q3 = I enjoy eating								0·889	
Q4 = I look forward to mealtimes								0·538	
Q29 = I eat slowly	1·67	4·8 %							0·907
Q25 = I am often last at finishing a meal									0·859
Q14 = I often finish my meals quickly									0·806
Q26 = I eat more and more slowly during the course of a meal									0·440

H: Hunger; FR: Food responsiveness; SR: Satiety responsiveness; EUE: Emotional under-eating; FF: Food fussiness; EOE: Emotional over-eating; EF: Enjoyment of food; SE: Slowness in eating.

* Factor loadings above 0·30 are presented. Extraction method: principal component analysis. Rotation method: Varimax with Kaiser Normalisation.

### Confirmatory factor analysis

Table 3 shows the results of confirmatory factor analysis and goodness-of-fit indicators. According to these findings, the seven-factor model provided a suitable model fit in the Turkish data. (chi-square/degrees of freedom = 2·137, root mean error of approximation: 0·061, comparative fit index: 0·884, and normed fit index: 0·850).

**Table 3:** Results of confirmatory factor analysis of the AEBQ-TR

Fit statistic	Model 1	Model 2	Original paper (7 factors)	Criteria
	7 factors (H + FR on a single factor)	8 factors (H + FR as separate factor)		
Chi-square/DF	2·137	2·235	4·5	< 5
RMSEA	0·061	0·063	0·061	< 0·08
CFI	0·884	0·875	0·896	≥ 0·80
NFI	0·850	0·806	0·871	h≥ 0·80

DF: Degrees of freedom, RMSEA: root mean square error of approximation, CFI: comparative fit index, NFI: normed fit index.

Table 4 indicates descriptive statistics (mean s d), internal validity (Cronbach’s alpha) and test–retest reliability for the eight-factor AEBQ-TR and the original eight-factor AEBQ validation study. The internal reliability of the AEBQ-TR shows adequate internal consistency of the questionnaire, with all Cronbach’s alphas greater than 0·70 except satiety responsiveness. Test–retest reliability was higher than 0·70 (0·95–0·98) for all subscales of the AEBQ-TR.

**Table 4:** Descriptive statistics and test–retest reliabilities of the AEBQ-TR and the original AEBQ validation

Appetite traits	AEBQ-TR (current study)						AEBQ (Hunot 2016)			
	Mean	sd	Internal reliability	95 % CI	Test–retest reliability	95 % CI	Internal reliability	95 % CI	Test–retest reliability	95 % CI
Hunger	3·15	1·04	0·74	0·69, 0·78	0·97	0·96, 0·98	0·75	0·73, 0·75	0·821	0·730, 0·881
Food Responsiveness	3·02	0·86	0·71	0·66, 0·76	0·97	0·95, 0·98	0·75	0·73, 0·75	0·871	0·805, 0·914
Emotional Over-eating	2·40	1·11	0·92	0·90, 0·93	0·98	0·97, 0·99	0·90	0·89, 0·91	0·732	0·596, 0·823
Enjoyment of the Food	3·89	0·88	0·83	0·79, 0·86	0·96	0·94, 0·97	0·86	0·84, 0·87	0·860	0·789, 0·907
Satiety Responsiveness	2·74	0·80	0·63	0·56, 0·70	0·95	0·93, 0·97	0·75	0·73, 0·78	0·865	0·797, 0·911
Emotional Under-eating	2·88	1·13	0·91	0·89, 0·92	0·98	0·96, 0·98	0·90	0·89, 0·91	0·772	0·656, 0·849
Food Fussiness	2·42	0·83	0·75	0·71, 0·79	0·97	0·96, 0·98	0·88	0·86, 0·89	0·907	0·860, 0·939
Slowness in Eating	2·79	1·04	0·80	0·76, 0·83	0·98	0·97, 0·99	0·88	0·87, 0·90	0·910	0·864, 0·940

### Associations between appetitive traits and BMI

The correlations between subscales are shown in Table 5. The ‘food approach’ subscales were, as expected, positively correlated with one another and generally negatively correlated with the ‘food avoidance’ subscales (Table 5), except for Hunger. Hunger was found to be related to Food Sensitivity, Emotional Over-eating and Food Enjoyment. Food Fussiness was not significantly associated with Slowness in Eating, but it was positively correlated with the ‘avoidance of food’ subscales.

**Table 5:** Pearson’s correlations and multivariable regression analyses between the AEBQ-TR scales and unadjusted and adjusted correlations with BMI in a Turkish sample

AEBQ	H	FR	EOE	EF	SR	EUE	FF	SE	BMI				
									Unadjusted (r)	Unadjusted (*β*)†	95 % Cl	Adjusted (*β*)†	95 % Cl
Food approach scales													
Hunger	1	0·617**	0·369**	0·315**	−0·077	−0·008	0·004	−0·034	−0·030	−0·032	–0·148, 0·085	−0·018	–0·119, 0·084
Food Responsiveness		1	0·410**	0·534**	−0·244**	−0·037	−0·218**	−0·187*	0·064	0·081	–0·060, 0·233	0·115	–0·008, 0·237
Emotional Over-eating			1	0·244**	−0·175**	−0·368**	0·061	0·028	0·154**	0·121	0·034, 0·207**	0·149	0·074, 0·223**
Enjoyment of the Food				1	−0·297**	−0·075	−0·249**	−0·221*	0·080	0·132	–0·052, 0·316	0·228	0·069, 0·387**
Food avoidance scales													
Satiety Responsiveness					1	0·332**	0·078	0·359**	−0·182**	−0·248	–0·397, –0·098**	−0·162	–0·295, –0·028*
Emotional Under-eating						1	−0·005	0·131*	−0·169**	−0·131	–0·216, –0·046**	−0·084	–0·160, –0·009*
Food Fussiness							1	0·011	0·147**	0·155	0·039, 0·272*	0·008	–0·099, 0·144
Slowness in Eating								1	−0·141*	−0·147	–0·264, –0·031*	−0·086	–0·190, 0·019

H: Hunger; FR: Food Responsiveness; SR: Satiety Responsiveness; EUE: Emotional Under-eating; FF: Food Fussiness; EOE: Emotional Over-eating; EF: Enjoyment of Food; SE: Slowness in Eating.

Adjusted for age, sex, education, and marital status.

* Correlation is significant at the 0·05 level (2-tailed).

** Correlation is significant at the 0·01 level (2-tailed).

† *β* (beta) values are unstandardised.

The relationships between BMI and the sub-dimensions of the scale are presented as three different models: (i) unadjusted relationships (Pearson’s correlations); (ii) unadjusted multivariate regressions; and (iii) multivariate regressions adjusted for gender, age, education level and marital status. Both higher Emotional Over-eating (*β* = 0·149, CI 0·074, 0·223, *P* < 0·001) and Enjoyment of the Food (*β* = 0·228, CI 0·069, 0·387, *P* < 0·001) were associated with higher BMI. Higher BMI was associated with lower Satiety Responsiveness (*β* = –0·162, Cl –0·295, –0·028, *P* < 0·001) and Emotional Under-eating (*β* = –0·084, Cl –0·160, –0·09, *P* < 0·001).

## Discussion

The AEBQ is a valid and reliable scale developed to assess adult appetitive traits. However, this comprehensive, valid and reliable measurement tool was not studied in Turkish. As a result, we performed the first Turkish validity and reliability analysis of the AEBQ, which is widely used in research and clinical practice.

The KMO value and the Bartlett sphericity test were used to determine the sample’s suitability for factor analysis. The value of the Bartlett sphericity test was determined to be statistically significant (*P* < 0·05) in this study, and the KMO value was greater than 0·60 (KMO = 0·83), both of which meet the criteria for performing factor analysis^(23)^.

According to factor analysis, the AEBQ-TR is a seven-dimensional scale with thirty-five items that explain 62·2 per cent of the variance. The factor loading of all scales was greater than 0·40 in this study. The factor load of all items in the original scale was higher than 0·30, and there were seven sub-dimensions^(14)^. The first scale, Hunger and ‘Food Responsiveness’, has nine items. The second scale is ‘Satiety Responsiveness’, which includes four items; the third is ‘Emotional Under-eating’, which includes five items; the fourth is ‘Food Fussiness’, which includes five items; the fifth is ‘Emotional Over-eating’, which includes five items; the sixth is ‘Enjoyment of Food’, which includes three items; and the final scale is ‘Slowness in Eating’, which includes four items. The original questionnaire’s construct validity is provided by the Turkish questionnaire. According to the literature, the structure determined by explanatory factor analysis should also be examined with confirmatory factor analysis for the scale’s validity and reliability^(24)^. In this study, the eight-factor structure of the AEBQ, including the Hunger scale, and the seven-factor model, which eliminates the Hunger scale, was tested with confirmatory factor analysis. It was determined that the confirmatory factor analysis fit indices of the seven-factor model had better model fit than the fit indices of the eight-factor model.

Moreover, the Australian and the Spanish studies stated that separating these scales provided the best model fit when there was a strong relationship between Hunger and Food responsiveness, but no relationship between hunger and weight^(15,17)^. Similarly, Hunot et al. (2016) found that the seven-factor structure improved model fit. Although Hunger and Food Responsiveness seem to be overlapping constructs, a validation study conducted in China determine that the eight-factor model including Hunger had a better model fit than a seven-factor model including ‘Food Responsiveness’ and ‘Hunger’ scales loaded on a subscale^(18)^. However, confirmatory factor analysis has not been performed to evaluate a model without the ‘Hunger’ scale suggested in the Chinese study. The findings of this study recommended that the ‘Hunger’ scale included in the original AEBQ be removed from future studies using AEBQ-TR. ‘Hunger’ items may be associated with internal states instead of a trait. Therefore, temporal factors such as the time of the last meal may have a greater influence on these items.

A measurement tool must be trustworthy in order to be valid^(25)^. Cronbach’s reliability coefficient, which measures the consistency of the scale’s sub-dimensions with the overall scale, was defined as reliable when it was greater than 0·60 in all sub-dimensions. In the initial study, Cronbach’s alpha values were reported in the range of 0·75–0·90 for all scales of the questionnaire^(14)^. The AEBQ-TR scale showed internal consistency characteristics that were similar to those of the original scale. The AEBQ-TR is quite reliable, as indicated by the reliability coefficient.

The test–retest methodology is yet another approach for assessing internal consistency^(26)^. In this study, the variation of the reliability coefficients of the scale sub-dimensions between 0·95 and 0·98 supports the test–retest reliability of the scale. Similarly, both the original validation and the Spanish study had test–retest reliability > 0·70^(14,17)^.

In general, food approach (H, FR, EOE and EF) and food avoidance (SR, EUE, FF and se) scales showed a positive correlation within themselves. In addition, mostly negative correlations were found between the food approach and avoidance scales. Unlike the original study, the statistically insignificant weak negative correlation between ‘Emotional Under-eating’ and ‘Hunger’in this study. In addition, contrary to the original study, weak positive relationships were between the ‘Food Fussiness’ and ‘Hunger’ scales, as well as the Emotional Over-eating and Slowness in Eating scales^(14)^. Similarly, the negative correlation between ‘Emotional Under-eating’ and ‘Hunger’ was also shown in the study in Chinese adults, but the other four studies found positive correlations^(15,17–19)^. Especially in the original study, the positive correlation between Emotional Under-eating and Hunger made it difficult to explain the Hunger scale^(14)^. In this study, the weak but negative correlation between ‘Emotional Under-eating’ and ‘Hunger’ scales is not surprising because the ‘Hunger’ scale aims to measure physiological hunger^(27)^. Therefore, these individuals, aware of the hunger-satiety signals, can manage their food intake in the face of their emotions^(28)^. Although the ‘Hunger’ scale is an important aspect of appetite that is not associated with emotional and restrictive situations, the inability to distinguish this expressed physiological hunger due to eating regulation or various eating attitudes (disinhibition, etc.) may explain the positive correlation between ‘Emotional Over-eating’ and ‘Hunger’^(14)^. Results related to the Hunger scale raise concerns about the validity of this scale and its use in Turkish society. Therefore, the ‘Hunger’ scale was combined with the moderately correlated ‘Food Responsiveness’ scale. In addition, the positive correlation between ‘Hunger’ and ‘Food Fussiness’ in this study may be because ‘Food Fussiness’ reflects selectivity in food selection, unlike other food avoidance scales that reflect increased sensitivity to satiety cues^(15)^. Furthermore, the unexpected positive correlation between ‘Emotional Over-eating’ and ‘Slowness in Eating’ may be due to the high eating speed of the majority of individuals in Turkish society.

Several studies showed a positive correlation between emotional eating and body weight^(29,30)^. In this study, there was only a relationship between higher Emotional Over-eating and higher BMI among the food approach features, while a correlation was found between higher Emotional Over-eating and Enjoyment of Food and higher BMI after adjusting age, gender, education and marital status. Various studies found similar results. Hunot-Alexander *et al.* (2022) found the relationship between higher ‘Emotional Over-eating’ and higher BMI after adjusting age, gender and data collection method. Mallan *et al.* (2017) determined a similar relation when adjusting age, gender, educational status, marital status and employment status ^17^. Unlike this study, Mallan *et al.* (2017) found a statistically significant higher BMI and lower ‘Hunger’, ‘Food Fussiness’, ‘Slowness in Eating’ relationship. On the other hand, Hunot *et al.* (2022) found a statistically significant relation between higher BMI and lower ‘Slowness in Eating’. Similar to this study, it is interesting that no relation was found between Food Responsiveness and BMI in both the Australian and Mexican studies. This may be due to the lack of awareness of the obesogenic environment.

Among the food avoidance scales, ‘Satiety Responsiveness’, ‘Emotional Under-eating’ and ‘Slowness in Eating’ scales are associated with lower BMI, while ‘Food Fussiness’ scale is associated with higher BMI. Similarly, a relationship was found between lower Satiety Responsiveness and Slowness in Eating and higher BMI in the Australian population after adjusting age, gender and data collection method and in the Mexican study after adjusting age, gender, educational status, marital status and employment^(15,17)^. These results support the hypotheses suggesting that obese individuals have higher ‘Food Fussiness’ features or that ‘Food Fussiness’ causes excessive energy intake due to the presence of palatable nutrients^(31,32)^. In various AEBQ validity studies, unrelated or negative/positive relationships were found between FF and BMI^(15,17–19)^. Results may differ depending on sampling (e.g. paediatric, bariatric surgery, etc.) or population habits. In addition, showing different results between ‘food approach’ and ‘food avoidance’ scales and BMI may be due to the fact that anthropometric measurements are based on self-reported in different studies^(14,18)^.

### Limitations

This study is a scale translated into Turkish to evaluate the AEBQ scale in adults. In addition, this scale may guide future research in Turkish adults. Although this article provides considerable data on AEBQ, it also has some limitations. Firstly, the results may not be applicable to the general population due to the cross-sectional design. Further studies with larger sample groups and other disease subgroups should be examined. Secondly, this study was carried out in a single city (adult Ankara residents), which may have resulted in bias. Future studies should assess its suitability for use in clinical and research settings (via clinical practice and/or other modes of administration such as face-to-face interviews) and with a wider range of populations, including clinical populations.

### Conclusion

The results of our study evaluating the relationship between AEBQ and BMI in adults in Turkey showed that AEBQ-TR is a valid and reliable scale for this population. AEBQ-TR has determined that appetitive properties related to the aetiology of weight change and especially obesity risk may be useful in research on adult population as in adolescents and children. Researchers recommend that further studies be conducted to examine population differences between AEBQ-TR scales and BMI in early and late adulthood. Also, repeating this scale in a larger sample and using longitudinal designs is recommended in future research.

## References

[1] Boutari C & Mantzoros CSA (2022) 2022 update on the epidemiology of obesity and a call to action: as its twin COVID-19 pandemic appears to be receding, the obesity and dysmetabolism pandemic continues to rage on. Metabolism 133, 155217. 35584732 10.1016/j.metabol.2022.155217 PMC9107388

[2] World Health Organization (2022) Who European Regional Obesity Report 2022. https://apps.who.int/iris/bitstream/handle/10665/353747/9789289057738-eng.pdf (accessed April 2022).

[3] Eurostat (2022) Overweight and Obesity – BMI Statistics. https://ec.europa.eu/eurostat/statistics-explained/index.php?title=Overweight_and_obesity_-_BMI_statistics (accessed May 2022).

[4] Llewellyn CH & Fildes A (2017) Behavioural susceptibility theory: professor jane wardle and the role of appetite in genetic risk of obesity. Curr Obes Rep 6, 38–45. 28236287 10.1007/s13679-017-0247-x PMC5359365

[5] Basolo A , Bechi Genzano S , Piaggi P et al. (2021) Energy balance and control of body weight: possible effects of meal timing and circadian rhythm dysregulation. Nutrients 13, 3276. 34579152 10.3390/nu13093276 PMC8470941

[6] Folkvord F & Hermans RC (2020) Food marketing in an obesogenic environment: a narrative overview of the potential of healthy food promotion to children and adults. Curr Addict Rep 7, 431–436.

[7] Sheikh AB , Nasrullah A , Haq S et al. (2017) The interplay of genetics and environmental factors in the development of obesity. Cureus 9, e1435. 28924523 10.7759/cureus.1435 PMC5587406

[8] Jacob R , Tremblay A , Fildes A et al. (2022) Validation of the adult eating behaviour questionnaire adapted for the French-speaking Canadian population. Eat Weight Disord 27, 1163–1179. 34185309 10.1007/s40519-021-01229-x

[9] Buckland NJ , Swinnerton LF , Ng K et al. (2021) Susceptibility to increased high energy dense sweet and savoury food intake in response to the COVID-19 lockdown: the role of craving control and acceptance coping strategies. Appetite 158, 105017. 33161044 10.1016/j.appet.2020.105017 PMC8580210

[10] Abdella HM , Farssi HO , El Broom DR et al. (2019) Eating behaviours and food cravings; influence of age, sex, BMI and FTO genotype. Nutrients 11, 377. 30759834 10.3390/nu11020377 PMC6412354

[11] Braden A , Emley E & Watford T (2019) Self-reported emotional eating is not related to greater food intake: results from two laboratory studies. Psychol Health 35, 500–517. 31455099 10.1080/08870446.2019.1649406

[12] Stunkard AJ & Messick S (1985) The three-factor eating questionnaire to measure dietary restraint, disinhibition and hunger. J Psychosom Res 29, 71–83. 3981480 10.1016/0022-3999(85)90010-8

[13] Van Strien T , Frijtiers JE , Bergers GP et al. (1992) The Dutch Eating Behavior Questionnaire (DEBQ) for assessment of restrained, emotional, and external eating behavior. Arch Pharm Res 15, 73–77.

[14] Hunot C , Fildes A , Croker H et al. (2016) Appetitive traits and relationships with BMI in adults: development of the adult eating behaviour questionnaire. Appetite 105, 356–363. 27215837 10.1016/j.appet.2016.05.024 PMC4990060

[15] Mallan KM , Fildes A , de la Piedad Garcia X et al. (2017) Appetitive traits associated with higher and lower body mass index: evaluating the validity of the adult eating behaviour questionnaire in an Australian sample. Int J Behav Nutr Phys Act 14, 1–8. 28938904 10.1186/s12966-017-0587-7 PMC5610469

[16] Hunot-Alexander C , Beeken RJ , Goodman W et al. (2019) Confirmation of the factor structure and reliability of the ‘Adult Eating Behavior Questionnaire’ in an adolescent sample. Front Psychol 10, 1991. 31636576 10.3389/fpsyg.2019.01991 PMC6788325

[17] Hunot-Alexander C , Arellano-Gómez LP , Smith AD et al. (2022) Examining the validity and consistency of the Adult Eating Behaviour Questionnaire-Español (AEBQ-Esp) and its relationship to BMI in a Mexican population. Eat Weight Disord – Stud Anorexia, Bulim Obes 27, 651–663. 10.1007/s40519-021-01201-9 PMC8933343 33966254

[18] He J , Sun S , Zickgraf HF et al. (2021) Assessing appetitive traits among Chinese young adults using the Adult Eating Behavior Questionnaire: factor structure, gender invariance and latent mean differences, and associations with BMI. Assessment 28, 877–889. 31328547 10.1177/1073191119864642 PMC7086485

[19] Zickgraf HF & Rigby A (2019) The Adult Eating Behaviour Questionnaire in a bariatric surgery-seeking sample: factor structure, convergent validity, and associations with BMI. Eur Eat Disord Rev 27, 91–104. 10.1002/erv.2628 PMC7052954 30039633

[20] Molitor SJ , Fox CK , Bensignor MO et al. (2021) Validity of the Adult Eating Behavior Questionnaire for adolescents treated in a weight management clinic. Int J Obes 45, 1086–1094. 10.1038/s41366-021-00778-6 33603129

[21] Guzek D , Skolmowska D & Głąbska D (2020) Appetitive traits in a population-based study of polish adolescents: validation of the Adult Eating Behaviour Questionnaire (AEBQ) and assessment within PLACE-19 study. Nutrients 12, 3889. 33352678 10.3390/nu12123889 PMC7766569

[22] Yurdugül H (2005) Using Content Validity & Indexes for Content Validity in Scale Development Studies. In XIV. National Educational Sciences Congress Pamukkale University Faculty of Education, 1–6. https://yunus.hacettepe.edu.tr/∼yurdugul/3/indir/PamukkaleBildiri.pdf (accessed April 2022).

[23] Kaiser HF (1974) An index of factorial simplicity. Psychometrika 39, 31–36.

[24] Burke Johnson LC (2019) Educational Research: Quantitative, Qualitative, and Mixed Approaches. USA: Sage Publications

[25] Taber KS (2018) The use of Cronbach’s alpha when developing and reporting research instruments in science education. Res Sci Educ 48, 1273–1296.

[26] Rattray J & Jones MC (2007) Essential elements of questionnaire design and development. J Clin Nurs 16, 234–243. 10.1111/j.1365-2702.2006.01573.x 17239058

[27] Karlsson J , Persson LO , Sjöström L et al. (2000) Psychometric properties and factor structure of the Three-Factor Eating Questionnaire (TFEQ) in obese men and women. Results from the Swedish Obese Subjects (SOS) study. Int J Obes 24, 1715–1725. 10.1038/sj.ijo.0801442 11126230

[28] Fuente González CE , Chávez-Servín JL , de la Torre-Carbot K et al. (2022) Relationship between emotional eating, consumption of hyperpalatable energy-dense foods, and indicators of nutritional status: a systematic review. J Obes 2022, 1–11. 10.1155/2022/4243868 PMC9132695 35634585

[29] Van Strien T , Herman CP & Verheijden MW (2009) Eating style, overeating, and overweight in a representative Dutch sample. Does external eating play a role? Appetite 52, 380–387. 19100301 10.1016/j.appet.2008.11.010

[30] Konttinen H , Haukkala A , Sarlio-Lä Hteenkorva S et al. (2009) Eating styles, self-control and obesity indicators. The moderating role of obesity status and dieting history on restrained eating. Appetite 53, 131–134. 19433123 10.1016/j.appet.2009.05.001

[31] Guss JL & Kissileff HR (2000) Microstructural analyses of human ingestive patterns: from description to mechanistic hypotheses. Neurosci Biobehav Rev 24, 261–268. 10714389 10.1016/s0149-7634(99)00079-2

[32] Schachter S (1971) Some extraordinary facts about obese humans and rats. Am Psychol 26, 129–144. 5541215 10.1037/h0030817

